# Improvements in obsessive–compulsive disorder and schizophrenia after left putaminal hemorrhage

**DOI:** 10.1111/pcn.13296

**Published:** 2021-09-06

**Authors:** Kohei Echizen, Eisuke Sakakibara, Motomu Suga, Kiyoto Kasai

**Affiliations:** ^1^ Department of Neuropsychiatry The University of Tokyo Hospital Tokyo Japan; ^2^ Department of Psychiatry Toranomon Hospital Kajigaya Kawasaki Japan; ^3^ Graduate School of Clinical Psychology Teikyo Heisei University Tokyo Japan

Obsessive–compulsive disorder (OCD) and schizophrenia are chronic and often treatment‐resistant disorders. Here, we report on a case of simultaneous improvements in OCD and schizophrenia symptoms following a putaminal hemorrhage. The patient was a 53‐year‐old, right‐handed, college‐educated woman with no family history of mental illness. At the age of 24, she developed obsessions with repetitive behaviors, such as checking documents for errors, confirming whether she had locked the door, confirming whether she had turned off the faucet, and washing her hands for more than 30 min for fear of contamination. She was aware of her irrationality but she could not stop herself from doing so. Following a visit to the hospital, she was diagnosed with OCD. At the age of 29, she developed auditory hallucinations that blame her and paranoid delusions that her family and surroundings were fake, that a ‘shady’ organization was targeting her, and that her food was being poisoned. A diagnosis of schizophrenia was made. Three months of inpatient treatment with 10 mg haloperidol and 162.5 mg chlorpromazine partially alleviated the hallucinations and delusions; however, it did not improve her OCD symptoms. After discharge, she received outpatient treatment and day care. Neither 6 months cognitive behavioral therapy nor up to 225 mg fluvoxamine relieved her OCD symptoms. The severity of the patient's illness resulted in her remaining unemployed after 32 years of age. The retrospectively assessed Yale‐Brown Obsessive Compulsive Scale, Positive and Negative Syndrome Scale, and Modified Global Assessment of Functioning scores for this period were 37, 85 (positive: 18, negative: 23, and general psychopathology: 44) and 21, respectively.

At the age of 51, she developed a left putaminal hemorrhage with intraventricular perforation that was treated conservatively (Fig. 1). After she regained consciousness the next day, no residual neurological symptoms remained, and, surprisingly, her OCD symptoms had completely resolved. Her psychotic symptoms also improved in both severity and frequency to the extent that she stated, ‘I sometimes feel like I'm hearing voices or being watched, but I don't care.’ The antipsychotics and fluvoxamine were tapered off with no relapses, to date. Her Yale‐Brown Obsessive Compulsive Scale, Positive and Negative Syndrome Scale, and Modified Global Assessment of Functioning scores were 0, 51 (positive: 10, negative: 18, general psychopathology: 23), and 68, respectively. She showed no noticeable signs of cognitive dysfunction or apathy, which are commonly seen after cerebral hemorrhages. Rather, she was able to perform household chores and leisure activities and go shopping, which she had been unable to do before.

**Fig. 1:**
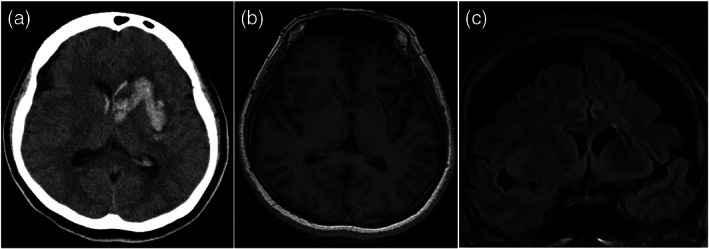
(a) The axial computed tomography image on the day of the onset of the putaminal hemorrhage. (b and c) The axial (b) and coronal (c) T1 weighted magnetic resonance imaging taken 2 years after the hemorrhage.

To our knowledge, this is the first‐time concurrent improvements in OCD and schizophrenia symptoms have been reported after a cerebral hemorrhage. This improvement cannot be attributed to the patient's possible inability to report existing symptoms, general cognitive dysfunction, or apathy that blunts daily activity as well as OCD and schizophrenia symptoms. The comorbidity of OCD and schizophrenia often occurs, and they share some pathophysiological similarities.^1^ As her comorbid OCD and schizophrenia occurred several years apart and responded to the medications differently, we proposed that they were separate disorders. However, the fact that both symptom complexes improved following the cerebral hemorrhage may have suggested an overlap in their neural basis.

Overactivity of the left putamen can be seen in both disorders.^2^, ^3^ In support of this hyperactivity being a contributing factor to the disorders, prior studies reported the improvement of OCD symptoms following left putaminal hemorrhage^4^, ^5^ One case report even described a resolution in auditory hallucinations and schizophrenic delusions following left putaminal hemorrhage.^6^ The hemorrhage may have ‘normalized’ the hyperactivity of the left putamen, which may in turn have improved the obsessive anxiety and delusions.

Additionally, the anterior limb of the internal capsule, which is adjacent to the putamen, may have also been damaged in this patient. The anterior limb of the internal capsule is a promising target site for ablative procedures such as anterior capsulotomy and deep brain stimulation for OCD.^7^ Psychosurgeries have also been reevaluated in recent years for schizophrenia.^8^ It is possible that the damage to this area worked like an incidental surgical intervention and thus also contributed to the improvement of both disorders.

Although our case may suggest the association of left putaminal hyperactivity with OCD and schizophrenia, the findings of previous studies are mixed as to the laterality of basal ganglia dysfunction underlying both disorders.^9^, ^10^ The case histories of patients with psychiatric disorders who later developed incidental focal cerebrovascular diseases give us valuable insights into the relationship between neural circuitry and clinical symptomatology.

## Disclosure statement

The authors declare no conflict of interest.

## Ethics statement

The study design was approved by the Ethics Committee of the University of Tokyo Hospital and conforms to the provisions of the Declaration of Helsinki.

## Informed consent

The study participant provided informed consent. We gave consideration to preserve anonymity of the patient.
